# Exposure Perception as a Key Indicator of Risk Perception and Acceptance of Sources of Radio Frequency Electromagnetic Fields

**DOI:** 10.1155/2015/198272

**Published:** 2015-07-01

**Authors:** Frederik Freudenstein, Peter M. Wiedemann, Tim W. C. Brown

**Affiliations:** ^1^Institute for Technology Assessment and Systems Analysis, Karlsruhe Institute of Technology, 10178 Berlin, Germany; ^2^School of Social Sciences, University of Wollongong, Wollongong, NSW 2522, Australia; ^3^Institute for Communication Systems Research, University of Surrey, Guildford, Surrey GU2 7XH, UK

## Abstract

The presented survey was conducted in six European countries as an online study. A total of 2454 subjects participated. Two main research questions were investigated: firstly, how does the cognitive, moral, and affective framing of radio frequency electromagnetic field (RF EMF) exposure perception influence RF EMF risk perception? Secondly, can the deployment of mobile phone base stations have greater acceptance with RF EMF exposure reduction? The findings with respect to the first question clearly indicated that the cognitive framed exposure perception is the main determinant of RF EMF risk perception. The concomitant sensitivity to exposure strength offers an opportunity to improve the acceptance of base stations by exposure reduction. A linear regression analysis supported this assumption: in a fictional test situation, exposure reduction improved the acceptance of base stations, operationalized as the requested distance of the base station from one's own home. Furthermore, subjects with high RF EMF risk perception were most sensitive to exposure reduction. On average, a 70% exposure reduction reduced the requested distance from about 2000 meters to 1000 meters. The consequences for risk communication are discussed.

## 1. Introduction

The fast-growing use of wireless communication technologies has stimulated concerns about the rise of public exposure to radio frequency electromagnetic fields (RF EMF) and fostered the prevailing societal discussion about potential human health risks. It is unsurprising that the public risk perception of RF EMF is at a constantly high level across Europe, especially for mobile phone base stations [1]. For instance, the Eurobarometer survey [2] shows that 46% of the people in the 27 European countries included are still fairly or very concerned about potential health risks of EMF. However, there are significant differences between the various countries (max: Greece 81% and min: Denmark 16%). Nevertheless, in most European countries the siting of base stations remains a controversial issue in inhabited areas, creating challenges for regulators and risk managers.

Several approaches in dealing with this resistance against the deployment of mobile phone base stations are proposed, for example, early public participation, improved risk communication, and measurement campaigns, which became an important topic for policy makers [3]. This is a difficult initial situation, especially when taking into account that even precaution can be seen as a confirmation of existing concerns [4]. Cousin and Siegrist [5] found that a lack of knowledge and understanding is associated with people's base station siting preferences. However, even more information and participation do not necessarily lead to a successful base station deployment process [6].

The low EMF exposure future networks (LExNet) project follows a different approach. It assumes that more acceptance of mobile phone base station deployments from the public could be achieved by reducing the exposure to RF EMF. LExNet expects that this reduction will result in a lower risk perception and therefore higher acceptance of EMF technologies [7]. Besides improving technical solutions for implementation, a social science section investigates the background of this relationship. In this line of thinking, we focus in this paper on three major issues: (1) RF EMF exposure perception, (2) RF EMF risk perception, and (3) acceptance of base station deployments. We will research how people perceive their personal exposure and explore whether cognitive, moral, or affective frames of exposure perception impact risk perception regarding various sources of exposure. In addition, we will analyze whether a reduction of RF EMF leads to more acceptance, using the example of mobile phone base stations (hereafter referred to simply as “base stations”).

## 2. Background

In a previous study (see [8]), we researched the impact of subjective knowledge about RF EMF exposure characteristics on the perception of health risks, that is, what people know about the impact of various exposure characteristics (e.g., “distance to the exposure source” and “duration of exposure”), regarding potential health effects. We found that this knowledge does influence people's risk perceptions. Better knowledge about the impact of these characteristics on potential health risks results in higher risk perception. However, the relationship between RF EMF exposure knowledge and RF EMF risk perception is complex and does not reveal how exposure perception is related to risk perception. We assume that the latter relationship depends on the frame in which level of EMF exposure is perceived. Three frames can be used: a cognitive frame, a moral frame, and an affective frame. In a cognitive frame, exposure is assessed regarding its intensity (“how intense is the exposure to the exposed person?”). In a moral frame, the moral rightness of the exposure is judged (“is being exposed fair?”); and in the affective frame, the feelings elicited by knowing that oneself is exposed play the dominant role (“is the exposure associated with good or bad feelings?”).

It seems reasonable to assume that people select one of these frames when they assess the risk of an exposure scenario. In other words, the cognitive frame may not necessarily be the dominant frame. People could focus on moral or affective aspects of exposure. This view is in line with the concept of intuitive toxicology developed by Slovic et al. [9]. According to this concept, lay people's risk judgments differ from expert ones because of their different conceptual structure. One example is the insensitivity of lay people to dose-effect relation, which is one of the basic principles in toxicology (see [10]). Furthermore, the Moral Foundation Theory [11] gives some hints about how RF EMF exposure could be viewed in an alternative frame. Moral issues, especially fairness, may play a crucial role. Furthermore, the knowledge that one is exposed to RF EMF may trigger negative feelings, which could amplify risk perceptions [12]. In this context Siegrist et al. [13] suggest that affect is an important factor in risk perception.

## 3. Research Aims

The study presented in the following examines fours questions in detail:

1. What do people believe about the level of RF EMF they are exposed to?
2. How is RF EMF risk perception influenced by cognitive, moral, and affective frames of RF EMF exposure perception?
3. Can the acceptance of base stations in one's own neighborhood be improved by RF EMF exposure reduction?
4. Does RF EMF risk perception mitigate the effects of exposure reduction on the acceptance of base stations?

While the first question is self-explanatory, the remaining three questions require elaboration. Whether exposure reduction results in more acceptance of telecommunication networks, especially base station deployments, depends in our view on the frame in which an RF EMF exposure situation is viewed. People's acceptance of base stations will be only a matter of RF EMF exposure reduction if a cognitive frame is applied. If RF EMF exposure is perceived solely within a moral or affective frame then information about any reduction of exposure will have little or no impact on the acceptance of base stations.

Furthermore, the dominant exposure perception frame might vary for different RF EMF exposure sources. Therefore, the impact of exposure frames has to be investigated across various sources of exposure.

However, even when a cognitive frame is applied in exposure perception, that is, when risk perception is based on people's belief about exposure levels, it is still open how much RF EMF exposure reduction is required in order to accept the deployment of a source of exposure. Therefore, we are interested in the effects of different exposure reductions. Furthermore, the acceptance of a base station deployment can be operationalized by the required distance from the base station to one's own home: the further the distance the higher the acceptance. This approach seems to be highly promising because people intuitively understand distance as “measure” for safety [14]. An additional advantage of asking for numerical distances is that numbers are less biased by subtle wording effects than verbal acceptance statements that are used in conventional rating scales.

## 4. Methods

The survey was conducted in August 2014 in six European countries as an online study by a professional survey company. A total of 2454 interviewees participated. After quality control 1809 respondents remained for analysis (German sample *n* = 274, French sample *n* = 243, Spanish sample *n* = 241, Portuguese sample *n* = 290, Romanian sample *n* = 276, Serbian sample *n* = 291, and UK sample *n* = 194).

The questionnaire consisted of 33 questions. All questions were translated into the languages of the participating countries and double-checked with retranslation back into English. An introduction to the survey informed the participants about the main research aims and what participation in the survey involves, including how anonymity of the survey is ensured. Some questions were introduced with additional information, for example, a technical background. In addition, we provided some background information about the LExNet project.

For investigating the exposure perception frames, we used questions guided by pictures, describing various exposure situations in a vivid way. We selected five scenarios: (1) exposure through using a mobile phone for calls, (2) exposure through laptop use, (3) exposure through using a WLAN (WiFi) router in a close position, (4) exposure through having a mobile communication mast (base station) on a roof close to one's home, and (5) exposure through the use of cell phones by others (the picture displayed a person using a mobile phone in public transport, sitting close to another passenger). The pictures were randomized in order of presentation.

We asked the subjects for how strong they consider the RF EMF exposure situation as well as for their affective and moral evaluation of the situation, all on a 5-point Likert scale. Furthermore, we asked the respondents to assess the level of danger of each of the five exposure situations (see Table 1).

Following a study from Wiedemann and Claus [15], we use the required distance from a base station to the person as a measure of acceptance by the person. Furthermore, the focus on base stations as a reference case for testing the impact of exposure reduction on acceptance of mobile telecommunication was chosen because of their thematic prominence in RF EMF risk perception. Risk perception research shows that base stations are the highest-ranked RF EMF risk source [8, 16].

Some of our demographic, political, and belief-related questions were derived from the “European Social Survey” [17]. The respondents were not forced to answer all questions. It was possible to skip questions or choose a “don't know” option.

## 5. Results

### 5.1. Characteristics of the Sample

The mean age of the participants was about 40 years, with 49.1% male and 50.9% female. The mean of respondents' education years of 15.2 indicates a sample of well-educated people, with a mode value (*n* = 268) of 12 years of education, that is, a level graduation equivalent to a high school diploma. Regarding the respondents' working situation in the last 7 days, the largest group (57% of the respondents) was in paid work (employees, self-employed, and working for your family business), 11.3% of the respondents were unemployed and actively looking for a job, and 9.0% were in education. In regard to the area in which they are living, more than 35% stated that they are residents in a big city and 15.1% in the suburbs of big cities. 34.7% said they live in a town or a small city, 13.4% in a country village, and 1.7% on a farm or home in the countryside.

### 5.2. Subjective Daily RF EMF Exposure Perception Level

**Figure 1:**
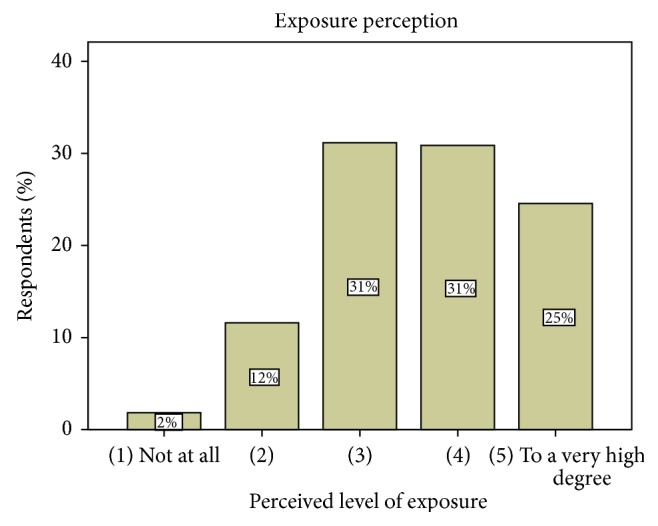
Perceived level of exposure (on a 5-point Likert scale from 1 “not at all” to 5 “to a very high degree”; question: “thinking about your daily life, to which degree do you think you are exposed to electromagnetic fields from electronic devices (like mobile phones, WiFi router) and base stations?”).

The general perceived level of RF EMF exposure is indicated by Figure 1. About 55% of the respondents believe that they have a high or very high exposure (4 or 5 on a 5-point Likert scale from 1 “not at all” to 5 “to a very high degree”). About 30% chose the midpoint of 3, and 14% claim that they have low exposure or are not at all exposed to RF EMF in their daily life (scored with 1 or 2).

These results show that many respondents believe that they are exposed to RF EMF to a high degree.

### 5.3. Affective, Moral, and Cognitive Exposure Frames and Risk Perception of Various RF EMF Exposure Sources

The question is, do people—when they assess the riskiness of an exposure situation—take exposure levels into account or are their risk perceptions rather influenced by moral or affective evaluation of RF EMF exposure situation? This question was researched for five exposure situations: using a mobile phone (MP) for calls, exposure through the use of cell phones by others, laptop use, using WLAN router in a close position, and exposure from a base station; see Table 2.

As demonstrated by Table 2 the base station is perceived as the most dangerous source out of the five (mean = 3.67 on the risk perception scale). It is also the source with the highest exposure perception (mean = 3.86), elicits the highest moral concerns (mean = 3.64), and has the highest negative affective scoring (mean = 3.59).

To analyze whether people's risk perceptions of various sources of EMF exposure are based on affective and moral frames or on a cognitive (taking exposure into account) frame, linear regressions were computed for all five exposure situations using risk perception as the dependent variable and the affective and moral evaluation as well as the subjective exposure perception as independent variables.  Table 3 indicates that the regression model provides a good explanation of the variance across all RF EMF exposure situations (*R* ^2^ from .672 for mobile phones to .822 for laptop use). A look at the beta values (*β*) reveals a robust pattern. Exposure frames seem to influence the risk perception to a high amount (mobile phone calls: *β* = .584, *p* = .000; WLAN close position: *β* = .629, *p* = .000; mobile phone use by others: *β* = .718, *p* = .000; laptop use on the lap: *β* = .670, *p* = .000; and base station: *β* = .711, *p* = .000).

Furthermore, as indicated by the beta values in the regressions, the influence of the affective frame on RF EMF risk perceptions is more or less negligible while the moral frame plays a role (*β*-value: *β* = .302 (*p* = .000) for mobile phones; *β* = .292 (*p* = .000) for WLAN close position; *β* = .222 (*p* = .000) for the use of a mobile phone by others; *β* = .269 (*p* = .000) for laptop use on the lap; and *β* = .208 (*p* = .000) for base stations). These findings point towards a consistent relationship. The RF EMF risk perceptions are mainly dependent on a cognitive frame, that is, on exposure strength perception. The higher the perceived RF EMF exposure, the higher the perceived risk. The same is true for moral concerns. The more the moral concerns involved, the higher the risk perception.

### 5.4. Effects of RF EMF Exposure Reduction on the Acceptance of Base Stations

Regarding the acceptance of RF EMF technologies we focused on the question of whether reductions of RF EMF exposure influence the acceptance of base station in one's own neighborhood. Specifically, we asked the respondents for the minimal distance (in meters) in which they would accept a base station close to their home for four different exposure conditions: (1) current exposure level without any reduction, (2) exposure level reduced by 30%, (3) exposure level reduced by 50%, and (4) exposure level reduced by 70%. For the analysis we excluded subjects answering a distance higher than 10 000 meters, that is, people who are in fundamental opposition to base stations (*n* = 70).

**Figure 2:**
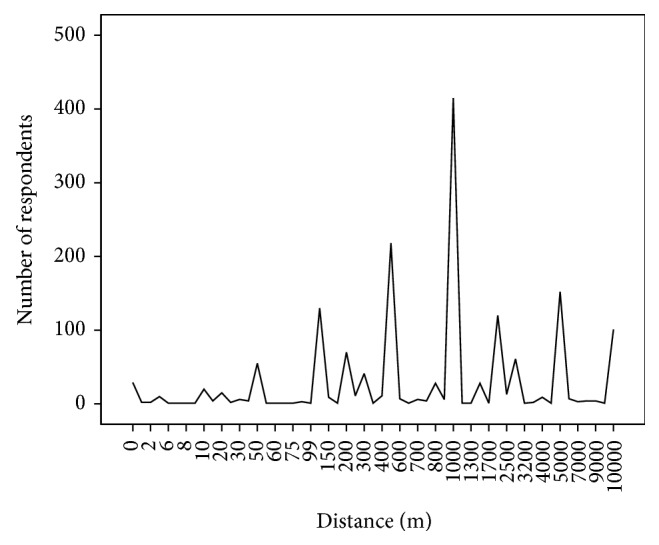
Accepted distances of base stations close to one's home in meter with 0% exposure reduction, indicated by numbers of respondents. For respondents with distance <10 000 meters (*n* = 1627). Question: “roughly at what distance (meters) would you accept a base station close to your home?”

Firstly, we have a look at the distribution of required distances for the exposure scenario (1), that is, the current exposure level. More than 25% (*n* = 415) of the participants selected exactly 1 km as the required distance to a base station for the exposure condition with 0% reduction. The next peak below this main effect can be found for distance of 500 meters (see Figure 2). The cumulated percentage of people who feel protected from exposure effects in a distance of up to 1000 meters to the base station amounts to 66% (*n* = 1072).

**Figure 3:**
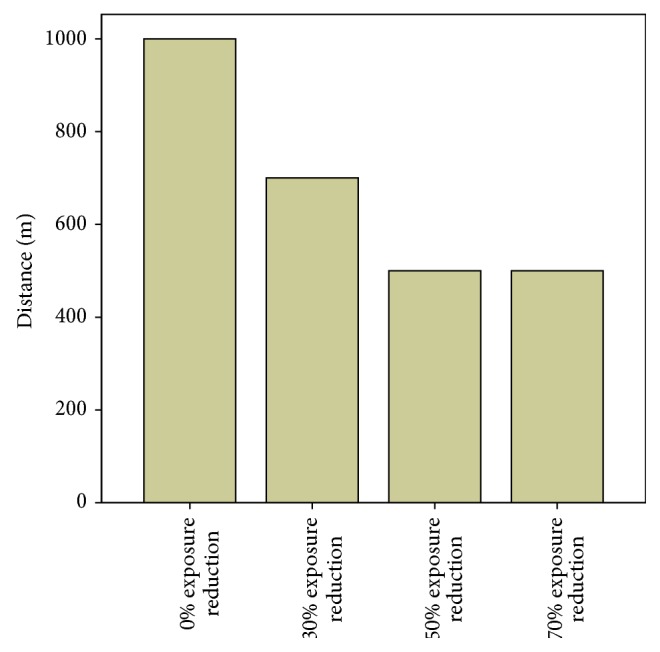
Median of distance in meters in which a base station close to one's home is accepted for 0%, 30%, 50%, and 70% exposure reduction. For respondents with distance <10 000 meters (*n* = 1627). Question: “roughly at what distance (meters) would you accept a base station close to your home?,” “… if the exposure was reduced by 30%?,” “… if the exposure was reduced by 50%?,” and “… if the exposure was reduced by 70%?”

In addition, we asked the subjects to consider the effects of fictional exposure reductions on their willingness to accept a base station deployment in their own neighborhood. The comparison between the four fictional exposure situations indicates a consistent picture: The higher the exposure reduction, the lower the distance in which a base station in the vicinity of one's home is accepted. While the median of the distance for the baseline exposure situation (0 = % reduction) is at 1000 meters, the median of the distance decreases to 700 meters for 30% exposure reduction, decreases to 500 meters for 50% exposure reduction, and finally remains at 500 meters for the highest exposure reduction of 70% (see Figure 3).

### 5.5. The Effects of RF EMF Exposure Reduction on the Acceptance of Base Stations in Dependency of Risk Perception

It seems reasonable to assume that RF EMF risk perception will influence the required distances across to the four exposure reduction scenarios. In order to test this hypothesis five risk perception groups are distinguished based on the scores for the perceived risk of base stations. The scores refer to one of above-mentioned pictured-guided scenarios that focused on the exposure by having a mobile communication mast (base stations) on a roof close to one's home.

**Figure 4:**
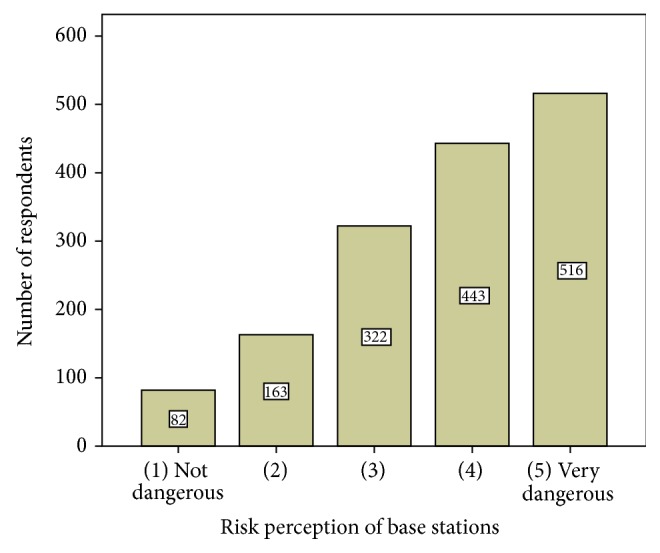
Frequency distribution of the scores for risk perception of base stations on a 5-point Likert scale (from 1 = “not dangerous” to 5 = very dangerous).

The frequency distribution of these risk perception scores is depicted by Figure 4. It indicates the strong tendency to evaluate the exposure from base stations as dangerous.

**Figure 5:**
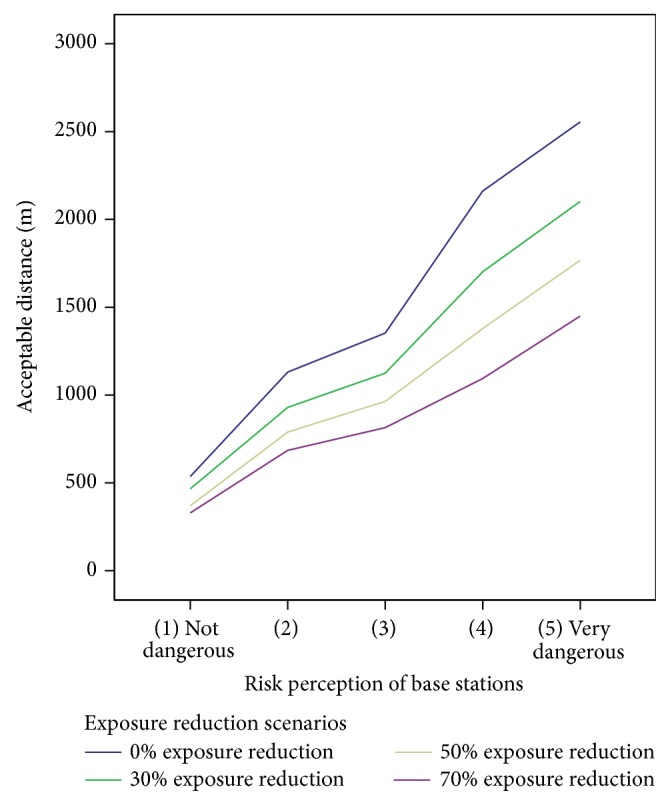
Effects of RF EMF exposure reduction on the acceptance of base stations in dependency of risk perception groups. Acceptance measured by the question “roughly at what distance (meters) would you accept a base station close to your home?,” “… if the exposure was reduced by 30%?,” “… if the exposure was reduced by 50%?,” and “… if the exposure was reduced by 70%?” Risk perception measured by the question “how dangerous do you consider living close to the building with the antennas,” on 5-point Likert scale from “1 = not dangerous” to “5 = very dangerous.” Meaning of lines: blue = 0% exposure reduction, green = 30% exposure reduction, yellow = 50% exposure reduction, and violet = 70% exposure reduction.

The different colored lines presented in Figure 5 display the required distances from the base station for the risk perception groups for the various degrees of exposure reduction (blue = 0% exposure reduction, green = 30% exposure reduction, yellow = 50% exposure reduction, and violet = 70% exposure reduction).

A general linear model with repeated measures was calculated using the different risk perceptions of base stations as “between subject factor” and the four exposure reduction scenarios as “within subject factor.” The required distance to one's own home was used as the dependent variable. The results show a significant main effect for the repeated factor exposure scenario (*F* = 148.884, *p* = .000 using Greenhouse-Geisser, empirical effect: *η* ^2^ = .089), as well as for the between subject effect for risk perception: *F* = 20.054, *p* = .000, *η* ^2^ = .050. The interaction between the main effect and the nonrepeated factor risk perception shows also a significant result: *F* = 12.160 and *p* = .000, using Greenhouse-Geisser, *η* ^2^ = .031. This means that the factors “exposure reduction” and “risk perception” have a statistically significant influence on the accepted distance from the base station and that the impact of the exposure reduction on this distance depends on the level of risk perception. The higher the risk perception, the higher the impact of exposure reduction.

Using the results from Figure 5, the effects of exposure reduction from 0% to 70% are outlined here. The difference for the five risk perception groups (Δ*d* = mean distance for 0% exposure reduction minus mean distance for 70% exposure reduction) is linearly increasing in the five risk perception groups: Δ*d*(1) = 207,24 meters, Δ*d*(2) = 445,17 meters, Δ*d*(3) = 537,77 meters, Δ*d*(4) = 1067,27 meters, and Δ*d*(5) = 1105,19 meters. The weakest effect is in the group with the lowest risk perception (Δ*d*(1)) and stepwise higher for the groups with the higher risk perception scores. However, in terms of the absolute distance (see Figure 5) the required distance increases with the level of risk perception.

Table 4 shows the Bonferroni adjusted post hoc test for calculated variance analyses between the independent variable risk perception and acceptance in meters (dependent variable) for every scenario to examine the differences between the risk perception groups.

The results indicate constant significant differences in the between group comparison among respondents with lower ((1), (2)) and higher risk perception ((4), (5)) (range from *p* = .000 to *p* = .010; except groups (2) and (4) for the 70% reduction scenario). This is also true for the differences between the risk perception scale midpoint of (3) and the groups of (4) and (5) (from *p* = .000 to *p* = .038).

Due to the fact that one of the requirements of the general linear model with repeated measurements is not fulfilled (Levene's test of equality of error variances) nonparametric test was calculated. The Friedman analysis of variances of ranks and the pairwise comparisons indicate significant differences between the four exposure reduction scenarios (*p* = .000 for all pairs of scenarios: 0%, 30%, 50%, and 70%). These results indicate that the higher the exposure reduction, the lower the required distance. In addition, Kruskal-Wallis tests prove that the requested distances depend on the level of risk perception for all exposure scenarios (*p* = .000 for all scenarios). The higher the risk perception, the higher the requested distance. These findings are in line with the above-reported results of the parametric analysis.

Finally, for exploratory reasons, we conducted several regression analyses with gender, age, and education as predictor variables and risk perception of various sources of EMF exposure as dependent variables (see Table 5).

The findings indicate various significant findings, especially for gender (mobile phone calls: *β* = .077, *p* = .002; laptop use: *β* = .085, *p* = .001; WLAN: *β* = .105, *p* = .000). It seems that female respondents have higher risk perceptions for 3 out of 5 sources of EMF exposure. However, these results suffer from the high levels of unexplained variance (*R* ^2^ = .007–.015). Therefore, they have to be interpreted with extreme caution.

## 6. Study Limitations

The chosen approach, based on a community sample, allows for testing of relationships between exposure perception, risk perception, and the effects of exposure reduction on acceptance of base station deployments. However, there are some limitations. The present study is based on a cross-sectional research design. This design has restrictions concerning the interpretation of statistical associations. Any causal interpretation of the established statistical associations needs further support from a randomized controlled study. Furthermore, one must be cautious in extrapolating these results to the general population. Firstly, the present study is based on an online survey that limits the scope for the generalization of the findings, as people without Internet access are not taken into account. Secondly, the country samples are not drawn randomly from the populations. Therefore, an extrapolation from our sample to the general population is restricted although the chosen sample is community based and represents a diversity of educational backgrounds. For the same reasons, conducting a cross-cultural analysis would not be practical.

## 7. Discussion

The presented research provides new insights into how people evaluate the risk of various RF EMF exposure situations. Firstly, our respondents believe that all considered exposure situations expose people to at least a medium level of RF EMF. The highest exposure is attributed to base stations and the lowest to the exposure caused by other persons' cell phone use. Our data show also that base stations tend to be associated with negative feelings. All other exposure situations do not elicit negative feelings. For moral concerns, the findings point at a more complex picture. All RF EMF exposure situations are associated with moral concerns, at least to a certain degree. Only base stations stimulate reasonably strong moral concerns. It must be noted that the type of moral concern was not specified in our questions. Therefore, we can only speculate about the moral dimension that the respondents refer to. According to Haidt's Moral Foundation Theory [11] we expect that harm is the key moral issue that people have in mind when they evaluate RF EMF exposure. This view is supported by the fact that many Europeans share the belief that RF EMF exposure might affect health [2]: two-thirds of the Europeans believed that their health is affected to some extent by high voltage power lines, mobile phone masts, and mobile phone handsets. There are two further interesting issues. Firstly, there is no EMF exposure situation that does not elicit moral concern. Even one's own use of cell phone is viewed to a certain degree as a moral issue. However, it does not mean that people intend to give up the usage of cell phones. One can believe that using a cell phone might be risky and still use a cell phone. Secondly and more importantly, risk communication meets its limits when moral reasoning is involved. Moral beliefs are not usually open to negotiations and deliberations. People are motivated to sustain their moral beliefs and discount arguments that challenge them [18]. The prevailing resistance to the siting of base stations might be rooted in the moral framing of siting controversies. Further research should explore whether—besides harm—other moral dimensions, such as fairness, are involved.

The determinants of intuitive EMF risk perception were analyzed by linear regressions across the four exposure situations. A consistent picture emerged: EMF risk perception is mainly affected by exposure perception and also to a certain degree by moral concerns. The affective evaluation of the exposure situations plays only a minor role. Both the high proportion of explained variances and high beta values for exposure perception in our regressions models clearly support this interpretation. In terms of risk communication, these findings provide a positive message. They suggest that, in principle, people should be sensitive to exposure reductions, simply because the level of exposure is an important factor when they evaluate EMF risks. Otherwise, any information about exposure reduction would have little or no impact.

This leads to the conclusion that a significant first step of risk communication is the framing of the EMF controversy. Communication should underline the importance of exposure issues and reduce the impact of moral frames. But even when the public acknowledges that exposure is the crucial point it does not necessarily mean that any reduction of RF EMF exposure—regardless of its amount—leads to reduced risk perceptions and to more acceptance of telecommunication technologies. The question “how safe is safe enough?” posed by Fischhoff et al. [19] years ago is the key. With regard to the RF EMF controversy this question reads as follows: “how much exposure reduction is required, in order to improve acceptance to a substantial degree?” To answer this question we tested four different exposure reduction scenarios regarding base stations, varying from 0% to 70% reduction. Here, a consistent picture emerged. The amount of exposure reduction significantly influences the distance from base stations required by the user. The higher the reduction, the shorter the required distance. This effect is the highest for people with elevated risk perceptions. These people are particularly sensitive to exposure reduction arguments. However, even a 70% exposure reduction reduces the required distance in the average only to about 1000 meters. In a densely populated city, the distance of base stations to the closed apartments, based on exclusion zones, is usually less than 50 meters [20]. Consequently, the requested mean distance in which a base station is accepted in the vicinity of people's homes does not correspond with the given circumstances.

For a communication strategy that builds upon exposure reduction, however, several points have to be taken into account that can only be briefly outlined here. Firstly, our previous study [8] demonstrated that lay people's knowledge about the impact of exposure characteristics on potential risks is important. It was found that lay people are aware of potential health risks depending on characteristics including the number of and duration of time intervals of exposure, which is particularly true in close proximity to a mobile source. The Interphone study from 2010 suggests that heavy cell phone users tend to have an increased risk of glioma at the highest exposure level, defined by the cumulative call time, that is, taking into account both duration and frequency of calls [21].

What seems to be lacking is the comparative view, that is, comparing the impact of these exposure characteristics for making trade-offs. Secondly, further research is needed in order to analyze the weight that lay people assign to the various RF EMF exposure conditions. Thirdly, built on this insight, one could explore which communication strategies are appropriate in order to strengthen informed judgments about the impact of various exposure sources. The dissemination of knowledge that explains that the assessment of the impact of the exposure of a base station requires trade-offs is especially important, for example, between exposure strength of the base station and the distance of the base station to the exposed people.

## Figures and Tables

**Table 1:** Questions on affective, moral, subjective exposure perception and risk perception of various exposure situations shown in pictures.

Question	Answer option
Affective evaluation: *“imagine you are the person depicted in the picture, what kind of feelings about exposure would you have in this situation?” *	5-point Likert scale from 1 = “very positive” to 5 = “very negative”

Moral evaluation: *“in your opinion, does the situation depicted by the picture elicit any moral concerns about exposure?” *	5-point Likert scale from 1 = “not at all” to 5 = “yes absolutely”

Subjective exposure perception: *“in your opinion, how strong is the exposure to the person in the above pictur*e*?” *	5-point Likert scale from 1 = “low” to 5 = “high”

Risk perception: *“how dangerous do you consider this situation to be for the person [placeholder describing scenario, e.g., using the laptop]? Please choose one of the following answers.” *	5-point Likert scale from 1 = “not dangerous” to 5 = “very dangerous”

**Table 2:** Means and variance of affective and moral evaluation, subjective exposure perception, and risk perception of various exposure situations, on 5-point Likert scale from 1 = “very positive” to 5 = “very negative” for affective evaluation; from 1 = “not at all” to 5 = “yes absolutely” for moral evaluation; from 1 = “low” to 5 = “high” for exposure evaluation; and from 1 = “not dangerous” to 5 = “very dangerous” for risk perception.

Evaluation of various sources of EMF exposure	*N*	Mean	Variance
Mobile phone (MP) calls			
Affective evaluation	1536	3.03	.862
Moral evaluation	1648	2.81	1.648
Subjective exposure perception	1643	3.34	1.472
Risk perception	1654	3.01	1.268
WLAN close position			
Affective evaluation	1546	2.90	.836
Moral evaluation	1630	2.67	1.411
Subjective exposure perception	1639	2.90	1.359
Risk perception	1627	2.76	1.296
MP use by others			
Affective evaluation	1547	3.05	.893
Moral evaluation	1659	2.59	1.383
Subjective exposure perception	1632	2.55	1.350
Risk perception	1640	2.44	1.231
Laptop use on the lap			
Affective evaluation	1572	2.91	1.025
Moral evaluation	1655	2.69	1.535
Subjective exposure perception	1637	2.91	1.482
Risk perception	1642	2.81	1.448
Base stations			
Affective evaluation	1629	3.59	1.423
Moral evaluation	1672	3.64	1.593
Subjective exposure perception	1657	3.86	1.389
Risk perception	1667	3.76	1.393

**Table 3:** Linear regression of affective, moral, and exposure evaluation on concerns about various sources of EMF exposure (risk perception), beta values indicated, *∗* = statistically significant (level .05).

Dependent variable risk perception of	*β*-values for situation evaluation			*R* ^2^
	Affective	Moral	Exposure	
Mobile phone (MP) calls	.092^*∗*^	.302^*∗*^	.584^*∗*^	.672
WLAN close position	.051^*∗*^	.292^*∗*^	.629^*∗*^	.756
MP use by others	.004	.222^*∗*^	.718^*∗*^	.790
Laptop use on the lap	.072^*∗*^	.269^*∗*^	.670^*∗*^	.822
Base station	.061^*∗*^	.208^*∗*^	.711^*∗*^	.811

**Table 4:** Bonferroni post hoc test for in between group differences among different risk perception groups and accepted distances to a base station for various exposure reduction scenarios. Question exposure reduction: “roughly at what distance (meters) would you accept a base station close to your home?,” “…if the exposure was reduced by 30%?,” “…if the exposure was reduced by 50%?,” and “…if the exposure was reduced by 70%?” Risk perception measured by the question “how dangerous do you consider living close to the building with the antennas?” on 5-point Likert scale from “1 = not dangerous” to “5 = very dangerous.” *∗* = statistically significant (level .05); *∗∗* = statistically significant (level .01).

Exposure reduction	RP groups	Mean distance (meters)	RP groups				
			(1) low	(2)	(3)	(4)	(5) high
0%	(1) low	536,55		.869	.103	.000^*∗∗*^	.000^*∗∗*^
	(2)	1130,37	.869		1	.000^*∗∗*^	.000^*∗∗*^
	(3)	1349,87	.103	1		.000^*∗∗*^	.000^*∗∗*^
	(4)	2161,26	.000^*∗∗*^	.000^*∗∗*^	.000^*∗∗*^		.140
	(5) high	2569,40	.000^*∗∗*^	.000^*∗∗*^	.000^*∗∗*^	.140	

30%	(1)	465,94		1	.157	.000^*∗∗*^	.000^*∗∗*^
	(2)	929,71	1		1	.001^*∗∗*^	.000^*∗∗*^
	(3)	1122,58	.157	1		.003^*∗∗*^	.000^*∗∗*^
	(4)	1702,04	.000^*∗∗*^	.001^*∗∗*^	.003^*∗∗*^		.049^*∗*^
	(5)	2102,26	.000^*∗∗*^	.000^*∗∗*^	.000^*∗∗*^	.049^*∗*^	

50%	(1)	369,45		1	.140	.000^*∗∗*^	.000^*∗∗*^
	(2)	789,11	1		1	.010^*∗*^	.000^*∗∗*^
	(3)	963,82	.140	1		.038^*∗*^	.000^*∗∗*^
	(4)	1378,79	.000^*∗∗*^	.010^*∗*^	.038^*∗*^		.022^*∗*^
	(5)	1767,50	.000^*∗∗*^	.000^*∗∗*^	.000^*∗∗*^	.022^*∗*^	

70%	(1)	329,30		1	.250	.003^*∗∗*^	.000^*∗∗*^
	(2)	685,19	1		1	.108	.000^*∗∗*^
	(3)	814,74	.250	1		.295	.000^*∗∗*^
	(4)	1093,99	.003^*∗∗*^	.108	.295		.017^*∗*^
	(5)	1449,78	.000^*∗∗*^	.000^*∗∗*^	.000^*∗∗*^	.017^*∗*^	

**Table 5:** Linear regression of age, gender, and education on concerns about various sources of EMF exposure (risk perception), beta values indicated, *∗* = statistically significant (level .05).

Dependent variable risk perception of	*β*-values for			*R* ^2^
	Gender	Age	Education	
Mobile phone (MP) calls	.077^*∗*^	−.030	.029	.008
WLAN close position	.105^*∗*^	−.001	.038	.012
MP use by others	.043	−.029	.000	.003
Laptop use on the lap	.085^*∗*^	−.071^*∗*^	.025	.015
Base station	.047	−.018	.016^*∗*^	.007
